# Building Digital Health Tools for Older Adults With Disabilities

**DOI:** 10.1093/geroni/igaa057.3177

**Published:** 2020-12-16

**Authors:** Fuad Abujarad, Sarah Swierenga, Chelsea Edwards

**Affiliations:** 1 Yale University, New Haven, Connecticut, United States; 2 Michigan State University, East Lansing, Michigan, United States; 3 Yale University School of Medicine, New Haven, Connecticut, United States

## Abstract

Adults with disabilities are more likely to be excluded from digital health research compared to those without. In this symposium, we will describe how user experience (UX) research can be used to successfully develop digital health tools that are both usable and acceptable by older adults with physical disabilities. Our framework and methodology are designed to provide older adults, age 60+ with hearing or visual impairments, the capability to use and adapt digital health tools (including tools that run on mobile devices like smart phones and tablets). Our approach includes one-on-one sessions to evaluate the ease of use and usefulness of digital health for older adults, including 6 blind, 6 deaf, 6 low vision, 6 hard of hearing. This approach allows us to increase the scope of the research participants and, eventually, end users to include adults with disabilities that we may not normally include in research study design.

